# Sequence diversity of apidaecin-like peptides arresting the terminating ribosome

**DOI:** 10.1093/nar/gkae567

**Published:** 2024-07-02

**Authors:** Weiping Huang, Chetana Baliga, Nora Vázquez-Laslop, Alexander S Mankin

**Affiliations:** Department of Pharmaceutical Sciences and Center for Biomolecular Sciences, University of Illinois at Chicago, Chicago, IL 60607, USA; Department of Pharmaceutical Sciences and Center for Biomolecular Sciences, University of Illinois at Chicago, Chicago, IL 60607, USA; Department of Pharmaceutical Sciences and Center for Biomolecular Sciences, University of Illinois at Chicago, Chicago, IL 60607, USA; Department of Pharmaceutical Sciences and Center for Biomolecular Sciences, University of Illinois at Chicago, Chicago, IL 60607, USA

## Abstract

The Proline-rich Antimicrobial Peptide (PrAMP) apidaecin (Api) inhibits translation by binding in the ribosomal nascent peptide exit tunnel, trapping release factors RF1 or RF2, and arresting ribosomes at stop codons. To explore the extent of sequence variations of the native 18-amino acid Api that allows it to preserve its activity, we screened a library of synthetic mutant Api genes expressed in bacterial cells, resulting in nearly 350000 peptide variants with multiple substitutions. By applying orthogonal negative and positive selection strategies, we identified a number of multi-substituted Api variants capable of arresting ribosomes at stop codons. Our findings underscore the critical contribution of specific amino acid residues of the peptide for its on-target function while significantly expanding the variety of PrAMPs acting on the terminating ribosome. Additionally, some of the tested synthesized multi-substituted Api variants exhibit improved antibacterial activity compared to that of the wild type PrAMP and may constitute the starting point to develop clinically useful antimicrobials.

## Graphical Abstract

**Graphical Abstract:**
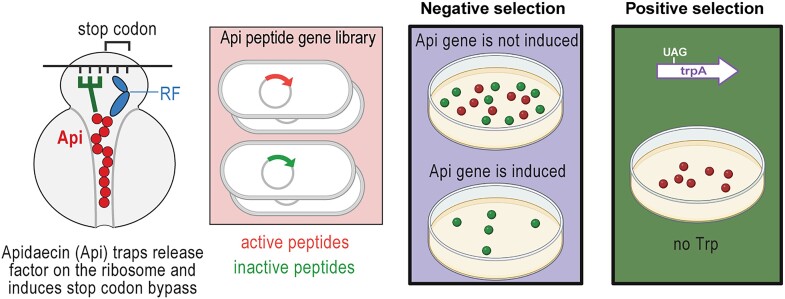

## Introduction

**Figure 1.**
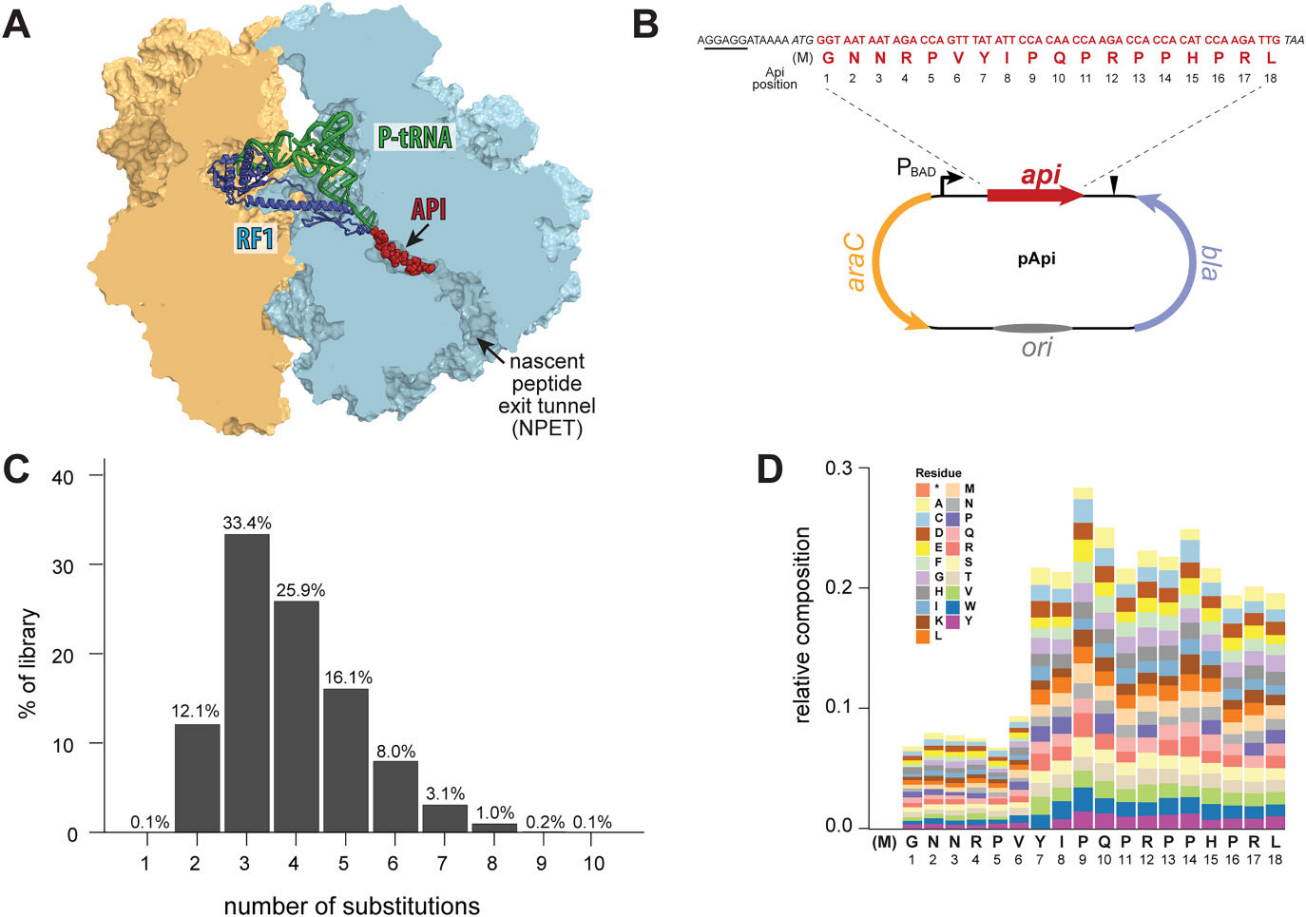
Library encoding Api peptide variants with multiple amino acid substitutions. (**A**) Api arrests post-release ribosomes at stop codons by binding in the NPET and trapping the RF and tRNA (based on the PDB ID 5O2R (8)). (**B**) Top, The sequence of the wt *api* gene. For construction of the expression plasmid library, a Shine-Dalgarno sequence (underlined), start codon and stop codon (both italicized) were added to the Api-coding sequence. Bottom, the structure of the expression plasmid for the mutant Api genes. The peptide gene (wild-type or mutant *api*) is controlled by the P_BAD_ promoter regulated by the AraC repressor. Transcription from the promoter is induced by arabinose and suppressed by glucose. Transcription terminator is indicated by a triangle. The ampicillin resistance gene *bla* and the replication origin (*ori*) are shown. (**C**) The distribution of the number of amino acid substitutions in peptides encoded in the unselected Api gene library. (**D**) The occurrence of mutations at each of the positions of the peptides in the unselected library; only amino acid substitutions (not the wt amino acid) are shown.

Proline-rich Antimicrobial Peptides (PrAMPs), produced by insects and mammals, inhibit growth of sensitive bacteria by targeting the ribosome (1–4). Like several known small molecule antibiotics, type I PrAMPs, interfere with initiation of translation (2,3,5,6). In contrast, Type II PrAMPs inhibit protein synthesis in a unique way – by acting at the translation termination stage. The first PrAMP characterized as Type II was the 18-amino-acid-long Apidaecin encoded in the genomes of honeybees (7,8). The predominant functional form of this PrAMP, Apidaecin 1b (Api) with the sequence GNNRPVYIPQPRPPHPRL, is processed from a longer precursor protein encoding multiple Api repeats (9). Released from the pre-pro-polypeptide, Api enters the cytoplasm of sensitive bacteria via specialized transporters such as SbmA (10). Once in the cytoplasm, Api diffuses up the vacant nascent peptide exit tunnel (NPET) of the ribosomes that have reached the stop codon, bound the release factor (RF1 or RF2), and released the completed protein (8,11). Api binds in the NPET in the extended conformation, with the C-terminus closely approaching the active site of the peptidyl transferase center (PTC) and the N-terminus extending down the tunnel (8,12) (Figure 1A). The side chain of the Api's penultimate arginine (R17) interacts with the A-site bound RF1 or RF2 (RF), whereas the carboxyl group of the C-terminal leucine (L18) closely approaches the 2′OH of the 3′terminal ribose of the P-site bound deacylated tRNA. Other Api amino acids establish multiple contacts with residues of the NPET, helping Api to lodge inside the ribosome (8,12). Api precludes the dissociation of the tRNA and RF, thereby arresting the ribosome at the stop codon. The sequestration of RFs in the Api-ribosome complexes leads, in turn, to the depletion of the pool of available RFs in the cell and triggers secondary events such as stop codon readthrough (8,13). More recently, a similar mode of action has been demonstrated for Drosocin (Dro), a PrAMP encoded in the fruit fly genome (14,15). While the sequence of Dro is somewhat similar to that of Api, the backbone trajectories of the two Type II PrAMPs in the NPET, as well as specific interactions with tunnel elements, significantly deviate between Api and Dro. These findings raise the possibility that peptides with significantly diverging sequences could form specific interactions in the NPET, trapping RFs and arresting ribosomes at stop codons. Such notion is further supported by limited studies of the biological activity of synthetic Api and Dro variants or their endogenously expressed genes showing that many single-substituted Api or Dro mutants remain active (15–22). It had remained unknown whether Type II PrAMPs can tolerate multiple mutations without losing their ability to bind in the NPET and trap the RFs.

In this paper, we used deep mutational screening powered by next-generation sequencing (NGS) to investigate the extent of sequence variation that can be tolerated by Api-like ribosome-arresting peptides. By using multi-substitution libraries of genetically encoded and endogenously expressed Api variants and applying both, negative and positive selection strategies, we identified a variety of peptides with Api-like activity thereby significantly expanding the diversity of protein sequences that are capable of arresting ribosomes at stop codons and trapping RFs. This approach also resulted in the identification of some multi-substituted Api variants with antibacterial activity comparable to, or even exceeding that of the wild type (wt) PrAMP.

## Materials and methods

### Bacterial strains and growth conditions

The *E. coli* strain CA244 (*lacZ125(Am), e14-, trpA49(Am), relA1, spoT1*) was obtained from *Escherichia coli* Genetic Stock Center. The *E. coli* BL21(DE3) electrocompetent cells were from Sigma. The following media and components were implemented for bacteria growth: lysogeny broth (LB), minimal M9 medium, Tryptic Soy Broth (TSB), Roswell Park Memorial Institute medium 1640 (RPMI-1640), Mueller Hinton Broth (MHB), Fetal Bovine Serum (FBS). For various experiments *E. coli* cells were grown either in LB, in 33% TSB (21), in M9 medium supplemented with 1% Glycerol, 1 mM MgSO_4_, 0.1 mM CaCl_2_ and 10 μg/ml thiamine, or in RPMI/Serum (RPMI-1640:MHB:FBS = 100:5:20) (23).

Liquid cultures were grown at 37°C on a rotary shaker set at 240 rpm. When necessary, media were supplemented with 0.2 mM isopropyl β-d-1-thiogalactopyranoside (IPTG), 40 μg/ml 5-bromo-4-chloro-3-indolyl-β-d-galactopyranoside (X-gal), 40 μg/ml tryptophan, and/or antibiotics: 30 μg/ml chloramphenicol (Chl), 100 μg/ml ampicillin (Amp) or 50 μg/ml kanamycin (Kan).

### Library construction

The DNA fragments (estimated diversity 10^10^) were synthesized by TWIST Bioscience to encode multiply substituted Api variants. Only one codon for each amino acid, predominant in the *E. coli* genomes, was used for the library construction. The codon context of the DNA library can be found in the (Supplementary Table S1). The peptide-coding sequence started with the ATG codon, preceded by Shine-Dalgarno (SD) sequence AGGAGG for ribosome binding, and ended with the TAA stop codon (Figure 1B). The peptide-coding gene was flanked with the sequences homologous to the insertion site in the pBAD18 cloning vector (24) (Supplementary Figure S1A). Upon introduction of the synthetic DNA into the pBAD18 vector, the expression of the peptide gene is controlled by the tightly regulated, arabinose-inducible P_BAD_ promoter (Figure 1B).

The original pBAD18 vector carries chloramphenicol resistance gene, *cat*, used for the selection and maintenance of the transformants. However, in our pilot experiments we noticed that cells transformed with the plasmids carrying unmutated Api gene (that we will refer to as ‘wt Api gene’) formed colonies of varying sizes on the selective Chl-containing plates. To alleviate this problem, the *cat* gene in pBAD18, originally inserted into the β-lactamase gene that confers resistance to Amp, was removed by inverse PCR and re-ligation of the plasmid. The resulting pBAD18A vector with the cloned wt Api gene produced uniformly sized colonies on the Amp/glucose plates and was used for all the subsequent experiments.

For generation of the plasmid library, the pBAD18A plasmid was cut with the restriction enzymes *Xho*I and *Xba*I and mutant Api gene library was introduced into it by Gibson assembly (25). The reaction mixtures were transformed into *E. coli* BL21 competent cells by electroporation. Transformants were recovered for 1 h at 37°C in Super optimal broth with catabolite repression (SOC) medium (26) and plated onto ten LB/agar plates supplemented with ampicillin and with 2% (w/v) glucose to repress the expression of the peptide genes. Approximately 10^7^ clones in total were obtained. All colonies were washed off the plates in LB, thoroughly mixed and, after addition of glycerol to 15% (v/v), aliquoted, snap frozen, and stored at -80°C serving as the library stock for subsequent experiments.

### Negative (depletion) selection

Prior to the library screening, pilot experiments were conducted to optimize arabinose concentration. To achieve this, *E. coli* BL21 cells transformed with the pApi plasmid carrying the wt Api gene were plated on LB/agar plates supplemented with Amp and increasing concentrations of arabinose in the 0.05–0.2% range. Cells carrying plasmid expressing the inactive (R17A) mutant of Api (11,22) were used as a negative control. Very limited growth of the wt pApi colonies was observed on the plates containing 0.05% arabinose (Supplementary Figure S2), whereas the cells expressing the R17A Api mutant formed large colonies (not shown). Subsequent library depletion experiments were carried out on plates containing 0.05% arabinose.

For the negative selection targeted at depleting the clones expressing active Api-like peptides, the 2 ml library stock containing a total of ∼2 × 10^9^ colony forming units (cfu) was thawed, diluted 3-fold in LB medium and allowed to recover for 30 min at 37°C. Subsequently, the cultures with the recovered cells were thoroughly mixed, and equal volumes (300 μl) were plated on ten LB/Amp plates supplemented with 2% glucose (for the total library) or ten LB/Amp plates supplemented with 0.05% arabinose (for the library depleted of clones expressing active Api variants). After incubation at 37°C for 16 h, clones were washed off the plates and cell suspensions were used for preparation of the total plasmid, resulting in the pApi(Glu) and pApi(Ara) plasmid preps. The depletion experiments were carried out in three independent replicates.

### Next-generation sequencing

For deep sequencing of the genes encoding Api-like peptides, the peptide-coding genes were PCR-amplified from the total pApi(Glu) and pApi(Ara) plasmids. Two 50 μl PCR reactions were carried out for each sample using Q5 DNA polymerase (New England Biolabs). The reactions were run in the manufacturer provided buffer, each reaction containing 20 ng of plasmid DNA and 10 pmol of primers Api_Lib_F_i5X and Api_Lib_R_i7X (Supplementary Table S2) which added specific bar codes to the individual samples. PCR amplification was carried out under the following conditions: 98°C, 30 s; 10 cycles of [98°C/10 s, 56°C/20 s, 72°C/10 s]; 72°C 120 s. The PCR products were size-fractionated and extracted from the denaturing 8% polyacrylamide gel. Bar-coded DNA libraries (Supplementary Figure S1B) were combined and sequenced on Illumina MiSeq after being spiked in with PhiX Control v3 library (Illumina) to compensate for sequence diversity of the sample.

### NGS data analysis

Sequencing has generated >350 million total reads for the three unselected samples and > 300 million total reads for the depleted library samples. The raw reads were trimmed to the size of the peptide gene and those containing internal ambiguous bases or being less than 60 bases in length were discarded. The nucleotide sequences were translated into amino acid sequences and the peptides lacking N-terminal methionine or deviating from the size of the expected sequence were eliminated. Peptides with <100 reads in each of the pApi(Glu) samples were also discarded. Subsequently, the filtered data underwent differential abundance analysis using the standard workflow of edgeR within the R/Bioconductor package (27). This analysis generated logFC (the log_2_-fold change of unique peptides between Glu and Ara samples), *q*-values (adjusted *P*-values with the FDR correction), and log_10_ CPM values (log counts per million), detailed in *Dataset 1*. A volcano plot was generated in R to visualize the differential abundance of unique peptides based on these parameters.

### Positive selection

To optimize the conditions for the positive selection of active peptides, *E. coli* CA244 electrocompetent cells were transformed with pApi or pApi(R17A) (inactive mutant). The transformed cells were plated on M9/X-gal/IPTG/Amp agar plates supplemented or not with 40 μg/ml tryptophan and containing varying concentrations of arabinose. Plates were incubated at 37°C for 1 day (with tryptophan) or 4 days (without tryptophan) in a tightly closed box with two wet paper towels to prevent plates from drying. Tryptophan-lacking plates containing 0.05% arabinose showed robust growth of blue colonies and this concentration of arabinose was then used in the library screening experiments.

For screening, the total library pApi(Glu) plasmids were transformed into *E. coli* CA244 electrocompetent cells. Following recovery of the transformed cells for 30 min at 37°C in LB, transformed cells were pelleted, washed with M9 medium and then plated on M9/X-gal/IPTG/Amp plates lacking tryptophan and containing 0.05% arabinose. After incubating plates at 37°C for 4 days, total of 1056 blue colonies were individually picked and cultured in LB/Amp overnight at 37°C in 96-well plates. Five microliter aliquots of overnight cultures of each clone were then pooled and used for plasmid isolation.

### Analysis of the NGS data for the positive selection screening

Library preparation and NGS were carried out as described above for the negative selection experiments. A total of 379,140 reads were generated which yielded 222 unique peptide sequences with read counts for individual peptide exceeding 70. The raw reads were merged, trimmed and filtered as described above for the negative selection experiments using Geneious Prime software (v. 2022.2). The unique peptide sequences resulting from the positive selection experiments can be found in the *Dataset 2* file).

### Peptide synthesis

Individual peptides, lacking N-terminal methionine, were synthesized by NovoPro (Shanghai, China). Peptides chosen from the negative selection experiments were synthesized at >90% purity with the N,N,N',N'tetramethylguanidino modification at the N-terminus to match the modification implemented in the Api137 derivative of Api (21). Peptides that emerged from the positive selection experiments were synthesized with unmodified N-terminus at >70% purity. The peptide stock solutions were prepared by dissolving them in water at 10 mM (adjusted for the content of the target product in the peptide preparation).

### MIC determination

The minimal inhibitory concentration (MIC) test was performed using the broth microdilution method, adapted from the previously described procedure (28). The MIC tests were conducted in triplicate either in LB, 33% TSB or RPMI/Serum media.

### Toeprinting analysis

Toeprinting experiments were performed as described previously (8,29). The modified *E. coli* gene *yrbA* (8) was used as a template for *in vitro* transcription/translation reactions using the PURExpress system (E6800L, New England Biolabs). Peptides were introduced into the reaction mixture from stock solutions to the final concentration of 50 μM.

### Stop codon readthrough assay

The dual RFP/GFP fluorescence reporter (15) based on the pRXG plasmid (30) was used to assess the ability of the synthetic peptides to inhibit cell growth and induce stop codon readthrough. Approximately 1 OD_600_ of *E. coli* BL21 cells transformed with the reporter plasmid were plated on M9/agar plates supplemented with 50 μg/ml of Kan. Once the surface of the plate was dry, 2 μl of 2 mM peptide solution was placed on top of the plate. Plates were incubated at 37°C for 16 h and then imaged on the ChemiDoc imager (BioRad) in the Cy3 and Cy2 channels (for RFP and GFP fluorescence, respectively). The images were processed and false colored using the ImageLab software (BioRad).

## Results

### Construction of the library of multiply substituted Api variants

To explore the diversity of peptides which, when translated in the cell, could bind in the NPET and trap RFs on the post-release ribosomes, we prepared a library of genes encoding peptides with predominantly two to six amino acid substitutions in the wt Api1b sequence. The library was designed to have a higher frequency of mutations in the C-terminal segment, which was previously shown to be functionally important for Api action (22). The peptide-coding sequence was amended with the start and stop codons and equipped with an optimized Shine-Dalgarno sequence to ensure its efficient translation in bacteria (Figure 1B). The highly diverse gene library with the theoretical complexity of >10^10^ variants was synthesized by massively parallel silicon-based DNA synthesis. Quality control NGS of the generated DNA fragment library confirmed its diversity and adherence to the desired properties (Supplementary Figure S1C).

The synthesized DNA library was introduced into the cloning vector, in which transcription of the gene is tightly controlled by the arabinose-inducible P_BAD_ promoter (Figure 1B). After the resulting pApi plasmid library was transformed into *E. coli* BL21 cells, ∼10^7^ clones were recovered on agar plates where expression of Api was repressed by glucose. NGS of the library recovered from the pooled clones showed its high diversity (349,199 unique sequences), the desired number of mutations (87% of the variants carried two to five mutations) (Figure 1C), the well-balanced rate of substitutions at all positions with the 19 alternative amino acids, and the targeted distributional bias of mutations towards the C-terminal segment of the encoded peptides (Figure 1D, Supplementary Figure S2). We therefore concluded that the plasmid expression library was amenable for the subsequent selection experiments.

### Phenotypic depletion identifies multiply substituted Api variants capable of arresting ribosomes at stop codons

**Figure 2.**
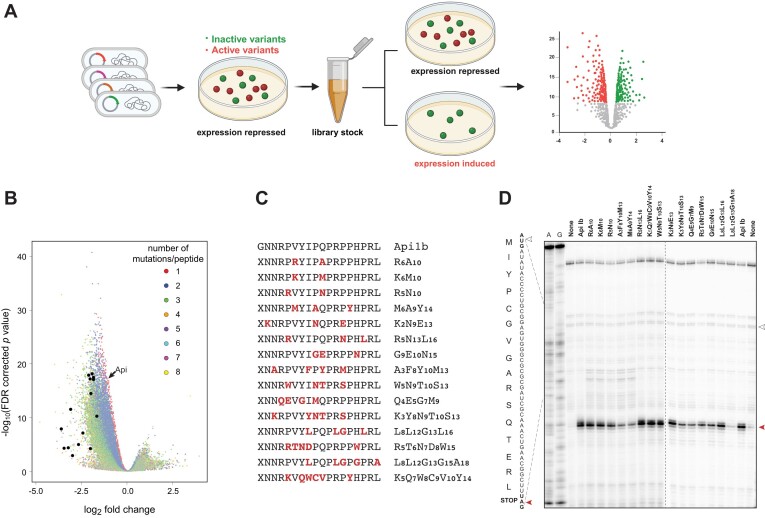
Identification of active peptides by the depletion selection. (**A**) The principle of the depletion selection approach: clones with plasmids encoding active peptides are depleted when grown under the induction conditions. (**B**) Volcano plot showing the change in the relative occurrence of clones expressing individual library peptides after the depletion selection. Wt Api is shown by an arrow. Peptides that were chemically synthesized and tested in biochemical and microbiological assays are indicated by arrowheads. (**C**) Sequences of the chemically synthesized peptides. Substituted amino acids are highlighted in red. Peptide names reflect the type and position of the mutated amino acid residues. All the peptides, excluding wt Api1b, were synthesized with *N*,*N*,*N*',*N*'-tetramethylguanidino-L-ornithine replacing the N-terminal glycine (indicated by ‘X’), because this modification has been shown to increase PrAMP’s uptake in microbiological testing (21). (**D**) *In vitro* toeprinting analysis of peptide-induced ribosome arrest at the stop codon of a model ORF. The white and red arrowheads indicate the toeprinting bands corresponding to the ribosomes at the start and stop codons of the ORF, respectively. Samples loaded onto the lanes marked as ‘None’ contained no added peptide. A- and G-specific sequencing lanes are indicated. The nucleotide sequence of the ORF, representing the modified and truncated *E. coli* gene *yrbA*, and the amino acid sequence of the encoded peptide are shown on the left. Note, that the toeprint band marking the leading edge of the ribosome is ∼16 nucleotides away from the first nucleotide of the P-site codon. Shown is a representative gel of two independent experiments that produced converging results. The uncropped gels can be found in Supplementary Figure S6.

Our first selection strategy was based on the observation that *in vivo* expression of wt Api or its functionally active singly substituted mutants is toxic to the cell (16–18,22). We therefore expected that the growth of *E. coli* cells expressing multiply substituted Api variants that retain the ability to arrest the ribosome and trap RFs would also be abolished or reduced. Accordingly, the negative selection involved plating the library of mutant Api gene clones on agar media supplemented either with glucose (where all the clones would grow) or with the inducer arabinose (where only clones expressing inactive Api variants would grow). Deep sequencing of the pulled clones recovered under these two conditions and computationally identifying the peptide sequences depleted upon *api* genes induction should yield the sequences of the active Api variants (Figure 2A).

Aiming to identify predominantly highly active peptides in the vastly diverse multi-substituted library, we optimized the concentration of arabinose inducer to reduce, but not completely abolish, the growth of the cells expressing wt Api (Supplementary Figure S3) and carried out the library depletion experiments under those conditions.

Approximately 10^9^ cells (a number that achieves a 100-fold coverage of the complexity of the clone library) were plated at high density on plates supplemented with glucose (no depletion control) or containing arabinose (depletion conditions) and then the plasmids from the entire population of clones that grew under each of these conditions were subjected to NGS. By comparing the relative occurrence of individual gene sequences in the control and depleted libraries, we were able to identify a variety of peptides whose expression decreased the representation of the corresponding clones in the population (Figure 2B). Out of the total of 349,199 peptide sequences in the unselected libraries, 15,654 variants were robustly depleted more than 2-fold [log_2_ fold change (FC) > 1; *q* value < 0.005] (Figure 2B, *Dataset 1*).

It was important to test whether depletion of the clones in the population is caused by the ability of the expressed peptides to trap ribosomes at stop codons. For this goal, we chose fifteen peptides featuring two to six amino acid substitutions, preferentially carrying the mutations in the C-terminal segment and characterized by a log_2_FC > 1.5 and *q* value < 0.005 (Figure 2C and *Dataset 1*). These peptides were chemically synthesized, and their activity was tested in a cell-free translation system by toeprinting, a technique that detects the position of the stalled ribosome on mRNA (29,31). Out of the fifteen tested peptides, fourteen, including one that carried six amino acid substitutions, could arrest the ribosome at the stop codon of a model gene (Figure 2D). This result not only validated our depletion selection strategy, but it also clearly showed that peptides with diverse sequences are able to arrest the terminating ribosome likely, similar to Api, by trapping RFs.

### Positive selection reveals multi-substituted Api-variants capable of inducing stop codon readthrough

A drawback of the depletion-based selection is that it does not immediately reveal the reason why expression of the peptide encoded by the mutant PrAMP gene is deleterious for the cell. Therefore, we conceived a library screening strategy which exploits stop codon readthrough, a known outcome of the Api-mediated RF trapping leading to depletion of available RFs (8,13,22,32). Specifically, we surmised that Api variant-mediated bypass of a premature stop codon in a conditionally essential gene would restore cell growth under non-permissive conditions and, therefore, could be used for the direct isolation of library clones expressing active peptides.

The *E. coli* strain CA244 harbors an amber (UAG) mutation in the *trpA* gene preventing its growth in the absence of tryptophan (33). In these cells, readthrough of the premature stop codon in *trpA* allows colony formation on tryptophan-lacking agar plates (34). The strain also carries an amber mutation in the *lacZ* gene encoding β-galactosidase whose activity, once restored by stop codon readthrough, can be visualized by the blue color of colonies on X-gal indicator plates. Accordingly, our positive selection strategy for identifying biologically active Api variants is based on plating the library on plates lacking tryptophan and containing low concentration of arabinose. We expected that moderate expression of active PrAMPs would induce stop codon bypass without killing the cells and result in formation of colonies that would turn blue if X-gal, the chromogenic substrate of β-galactosidase, is present. The use of this second (*lacZ*) reporter allows distinguishing colonies growing in the absence of tryptophan due to stop codon readthrough from *trpA* mutational revertants.

**Figure 3.**
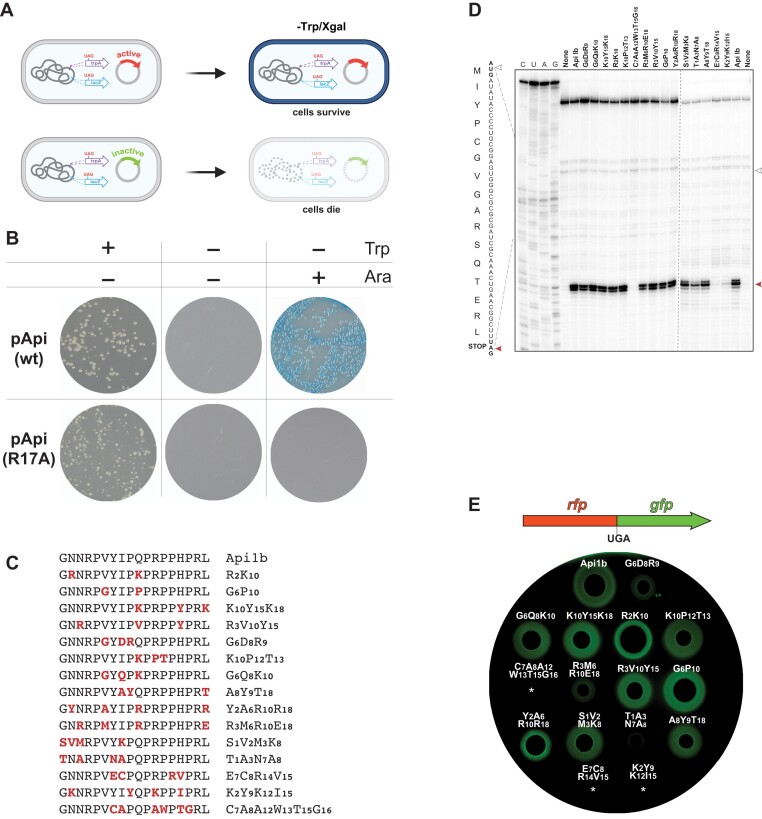
Identification of active peptides by the positive selection. (**A**) The principle of the positive selection approach: clones expressing subinhibitory concentrations of the active peptides can form colonies on the tryptophan-lacking plates. (**B**) Api-induced premature stop codon readthrough in the *trpA* and *lacZ* genes of the *E. coli* strain CA244 leads to formation of blue-colored colonies on the indicator agar plate lacking tryptophan. The CA244 cells were transformed with the plasmids encoding either wt Api or its inactive R17A mutant (21,22). Moderate expression of Api is induced by low (0.05%) concentration of arabinose. (**C**) Sequences of the chemically synthesized peptides identified by the positive selection. Altered amino acids are highlighted in red. Peptides were synthesized without additional N-terminal modifications. (**D**) Toeprinting analysis of the peptide-induced ribosome arrest at the stop codon of the modified *yrbA* ORF. The white and red arrowheads indicate the toeprinting bands corresponding to ribosomes at the start and stop codons of the ORF, respectively. Samples loaded onto the lanes marked ‘None’ contained no added peptide. Sequencing lanes are indicated. The uncropped gels can be found in Supplementary Figure S6. (**E**) Drop-diffusion assay reveals the activity of the synthesized peptides. Top, the scheme of the reporter construct with the in-frame fused *rfp* and *gfp* genes separated by the UGA stop codon. Peptide-induced stop codon bypass allows for GFP expression. Bottom, *E. coli* BL21 cells transformed with the reporter plasmid were plated on agar plates and 2 μl drops of 2 mM peptide solutions were spotted on the agar surface. Plates were imaged for the GFP fluorescence. Clearing zones, visualized as black circles, indicate no cell growth due to the antibacterial activity of the peptides. The green halos indicate GFP expression when subinhibitory concentrations of the peptides lead to stop codon readthrough. White asterisks show the sites of application of peptides that did not have antimicrobial activity. Shown are representative gels (**C**) or image of a plate (**D**) of two independent experiments that produced converging results.

We validated the approach by plating the CA244 cells transformed with the wt pApi plasmid on minimal medium plates lacking tryptophan and containing X-gal and varying concentrations of arabinose. While no colonies grew on plates with 0.5% arabinose, likely due to the detrimental effect of high expression of Api upon cell growth (see Supplementary Figure S3), blue colonies formed on plates with lower arabinose concentration (0.05%) (Figure 3A), indicating that moderate levels of Api expression can stimulate stop codon bypass in both *trpA* and *lacZ* mutant genes.

We then used these optimized conditions to identify the library clones expressing peptides capable of stimulating stop codon bypass. The *E. coli* CA244 cells transformed with the pooled library plasmid were plated on the tryptophan-lacking and X-gal supplemented selection plates and a total of 1,056 blue colonies were manually picked and individually grown in 96-well plates.

After NGS sequencing of the pooled plasmid from these clones, 222 unique sequences (including wt Api) were identified comprising peptides with one, two, three, four, and six substitutions (*Dataset 2*). Fifteen peptides with varying number of mutations (Figure 3B) were chemically synthesized and tested in *in vitro* and *in vivo* assays (Figure 3C-D). The *in vitro* toeprinting assay showed that twelve of the tested peptides readily stalled ribosomes at the stop codon (Figure 3C). To directly evaluate whether the synthetic peptides promote *in vivo* stop codon bypass we used *E. coli* BL21 cells transformed with a reporter plasmid in which the fused *rfp* and *gfp* genes are separated by the UGA stop codon (15,30). In the drop-diffusion test, eleven out of the twelve peptides that showed activity in the toeprinting assay generated a halo of GFP-expressing cells (Figure 3D), confirming the ability of these peptides to induce stop codon readthrough. [Noteworthy, the few peptides with sufficient antibacterial activity (Table 1) that originated from the depletion selection experiments could also induce stop codon readthrough (Supplementary Figure S4)]. The results of the positive selection experiments corroborated the conclusion that emerged from the depletion selection: namely, that peptides deviating in multiple amino acid residues from the wt Api sequence are able to interact with the ribosome, arresting it at stop codons and trapping the release factors.

**Table 1.** Minimal inhibitory concentrations of the synthesized peptides against *E. coli*, BL21 cells

Peptides identified by depletion selection				
		MIC (μM)		
Peptide	Sequence^a^	RPMI/Serum	TSB	LB
Api 137	XNNRPVYIPRPRPPHPRL	0.0625	0.25	4
Api Ib	GNNRPVYIPQPRPPHPRL	4	0.5	16
R_6_A_10_	XNNRPRYIPAPRPPHPRL	0.0625	0.5	4
K_6_M_10_	XNNRPKYIPMPRPPHPRL	1	0.5	8
R_5_N_10_	XNNRRVYIPNPRPPHPRL	1	8	32
A_3_F_8_Y_10_M_13_	XNARPVYFPYPRMPHPRL	8	16	>64
M_6_A_9_Y_14_	XNNRPMYIAQPRPYHPRL	16	16	>64
R_5_N_13_L_16_	XNNRRVYIPQPRNPHLRL	>64	>64	>64
K_5_Q_7_W_8_C_9_V_10_Y_14_	XNNRKVQWCVPRPYHPRL	>64	>64	>64
W_5_N_9_T_10_S_13_	XNNRWVYINTPRSPHPRL	>64	>64	>64
K_2_N_9_E_13_	XKNRPVYINQPREPHPRL	>64	>64	>64
K_3_Y_8_N_9_T_10_S_13_	XNKRPVYYNTPRSPHPRL	>64	>64	>64
Q_4_E_5_G_7_M_9_	XNNQEVGIMQPRPPHPRL	>64	>64	>64
R_5_T_6_N_7_D_8_W_15_	XNNRRTNDPQPRPPWPRL	>64	>64	>64
G_9_E_10_N_15_	XNNRPVYIGEPRPPNPRL	>64	>64	>64
L_8_L_12_G_13_G_15_A_18_	XNNRPVYLPQPLGPGPRA	>64	>64	>64
L_8_L_12_G_13_L_16_	XNNRPVYLPQPLGPHLRL	>64	>64	>64
**Peptides identified by positive selection**.				
		**MIC (μM)**		
**Peptide**	**Sequence**	**RPMI/Serum**	**TSB**	**LB**
Api Ib	GNNRPVYIPQPRPPHPRL	4	0.5	16
R_2_K_10_	GRNRPVYIPKPRPPHPRL	0.5	0.25	2
Y_2_A_6_R_10_R_18_	GYNRPAYIPRPRPPHPRR	0.5	1	4
K_10_Y_15_K_18_	GNNRPVYIPKPRPPYPRK	2	2	8
R_3_V_10_Y_15_	GNRRPVYIPVPRPPYPRL	2	2	8
G_6_P_10_	GNNRPGYIPPPRPPHPRL	4	0.5	8
G_6_D_8_R_9_	GNNRPGYDRQPRPPHPRL	4	8	16
K_10_P_12_T_13_	GNNRPVYIPKPPTPHPRL	8	1	64
G_6_Q_8_K_10_	GNNRPGYQPKPRPPHPRL	16	0.5	32
R_3_M_6_R_10_E_18_	GNRRPMYIPRPRPPHPRE	16	8	>64
C_7_A_8_A_12_W_13_T_15_G_16_	GNNRPVCAPQPAWPTGRL	>64	>64	>64
S_1_V_2_M_3_K_8_	SVMRPVYKPQPRPPHPRL	4	0.5	16
T_1_A_3_N_7_A_8_	TNARPVNAPQPRPPHPRL	16	8	64
A_8_Y_9_T_18_	GNNRPVYAYQPRPPHPRT	8	4	32
E_7_C_8_R_14_V_15_	GNNRPVECPQPRPRVPRL	>64	>64	>64
K_2_Y_9_K_12_I_15_	GKNRPVYIYQPKPPIPRL	>64	>64	>64

^a^ ‘X’ stands for *N*,*N*,*N*’*N*’-tetramethylguanidino-L-ornithine described in (21), which replaces the N-terminal glycine in all peptides but wt Api 1b.

### Trends in sequence and physicochemical properties of api-like peptides conducive to their ribosome-targeting activity

**Figure 4.**
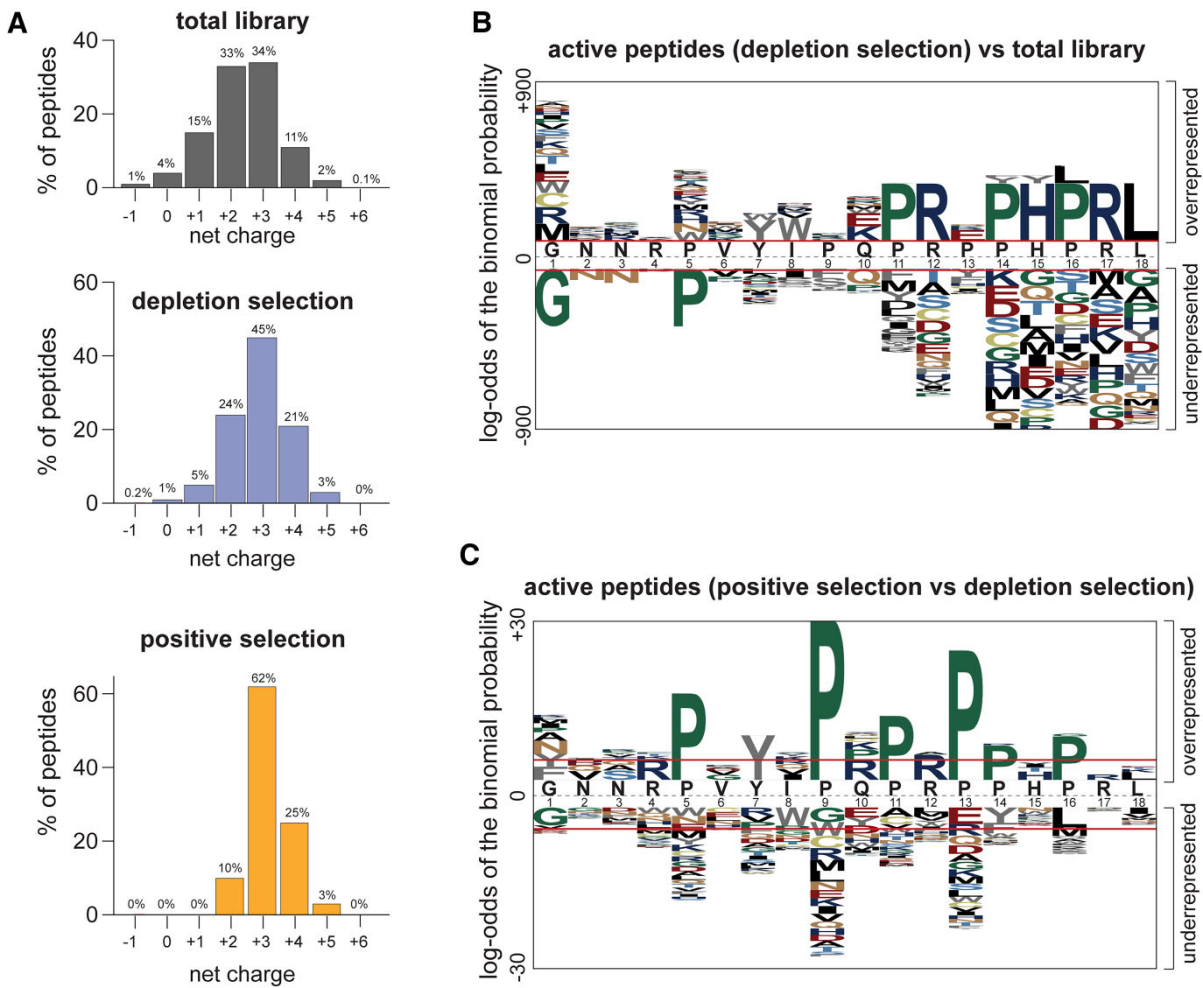
Compositional and sequence trends in the active peptides selected from the library. (**A**) Distribution of the overall net charge among peptides encoded in the unselected library (top panel), active peptides identified by the depletion selection (middle panel), and active peptides identified by the positive selection. (**B**) pLogo analysis of amino acid enrichment at individual positions of the active peptides identified by the depletion selection in comparison with the unselected peptides. Preferred amino acids appear in the upper panel; the amino acids that are counter-selected in the active peptides appear in the bottom panel. (**C**) pLogo analysis of amino acid enrichment at individual positions of the active peptides identified by the positive selection in comparison with the peptides identified by the depletion selection.

The vast diversity of the mutant Api gene library and the high number of putatively active peptides found via the negative and positive selection approaches allowed identifying the composition and sequence trends of the peptides with ribosome-targeting activity. We observed that contrasting the broad overall net charge distribution of the peptides in the unselected library (ranging from –1 to +6), the net charge of the active PrAMPs falls within the narrow range of +2 to +4 (Figure 4A). It appears that a moderate positive charge benefits the peptides’ ability to form specific interactions with the negatively charged NPET.

The most interesting insights into the requirement for the PrAMPs’ activity came from the examination of their sequences. We used pLogo analysis (35) to identify the features that distinguish the active PrAMPs from those in the unselected library (Figure 4B and Supplementary Figure S5). pLogo plot of the peptides from the depletion selection shows that seven out of the eight C-terminal residues of Api poorly tolerate most substitutions (Figure 4B). The nearly complete lack of mutations of amino acids P_11_, R_12_, R_17_ and L_18_ reflect their critical functional significance for interacting with the ribosome-bound RF and tRNA (R_17_ and L_18_, respectively), or with the NPET elements (P_11_, R_12_) (8). Several other residues in the C-terminal segment (e.g. P_14_, H_15_, P_16_) allowed for some replacements, but the Api's wt amino acids at these positions are nevertheless strongly preferred. Only mutations of P_13_, in particular substitutions with glutamic acid (E), appear to be rather forgiving. In contrast to the conservation of the C-terminal segment, the sequence trends in the N-terminal half of the peptide were dramatically different. Here, many alternative amino acids are not only as good as the residues present at these positions in wt Api, but some of the wt amino acids are even counter selected in the active peptides. The prevalence of substitutions over wt are particularly evident for G_1_ and P_5_, but it is also rather noticeable for several other N-terminal residues. Thus, for example, replacements of Q_10_ with the charged amino acids or of I_8_ with an aromatic tryptophan is conducive to the PrAMP’s activity.

Although the number of active peptides identified by positive selection was much smaller (222 vs 15,654), similar trends (e.g. conservation of the Api's C-terminal sequence and counterselection of some of the wt N-terminal amino acids) could be also observed in the pLogo plot of these PrAMPs (Supplementary Figure S5). Nevertheless, some characteristics of the PrAMPs that emerged from the two orthogonal selection strategies seem to deviate (Figure 4C). Peptides capable of inducing stop codon bypass (the main criteria of the positive selection) exhibit strong preference for retaining all of the Api proline residues (Figure 4B-C). In addition, the peptides of the positive selection also frequently have Q_10_ replaced with positively charged arginine or lysine residues, but not with the negatively charged glutamate, often found in this position in the active peptides identified by depletion selection (compare Figure 4B, C and Supplementary Figure S5). Such subtle, but important differences in the sequence trends between two groups of active peptides suggest that inhibition of cell growth by the endogenously expressed Api-like PrAMPs may involve aspects other than the RF trapping which leads to stop codon bypass.

### Enhanced antimicrobial potency of multiply substituted Api variants

While the primary goal of our study was to identify the spectrum of the endogenously expressed Api-like peptides with the ribosome targeting activity, we could not ignore the fact that wt Api is a potent antimicrobial with prospects to be developed into a clinically-relevant antibiotic (20,21,36). For its antibiotic activity wt Api needs to be imported into the bacterial cell by the SbmA transporter. Therefore, we were prepared to the possibility that some of the peptides, identified in our selection experiments as inhibitory when produced in the bacterial cytoplasm, would not be transported into the cell when added exogenously. Nevertheless, we were encouraged by the results of the drop-diffusion readthrough assay, where some of the peptides produced a distinct zone of cell growth inhibition, which for some of the PrAMPs even exceeded the size of the clearing zone produce by wt Api (Figure 3D).

In order to quantitatively assess the antimicrobial activity of the synthesized peptides identified by both, the negative and positive selection strategies, we determined their minimal inhibitory concentrations (MICs) for the *E. coli* BL21 cells in three different media: LB, as a standard laboratory medium, 33% TSB, as a medium in which PrAMPs show increased antibacterial properties (21), and in the more therapeutically relevant RPMI/serum media (23) (Table 1). Importantly, several of the peptides that originated from positive selection (e.g. R_2_K_10_, Y_2_A_6_R_10_R_18_, K_10_Y_15_K_18_, R_3_V_10_Y_15_) were more active than wt Api in LB and RPMI/serum media and few others were as active as wt Api (Table 1). A notably smaller fraction of the peptides that came from the depletion selection could interfere with cell growth. Nevertheless, some of them (e.g. R_6_A_10_, K_6_M_10_ and R_5_N_10_) were characterized by the MIC values comparable to those of wt Api. It appears, therefore, that the positive selection strategy is more prone to identifying biologically active peptides in comparison with the negative selection approaches. Furthermore, it became apparent that some multiple substitutions can even improve the antibacterial properties of the Api-like peptides.

## Discussion

The unique mode of action of Api, which has the ability of trapping RF1/2 on the ribosome raised the question whether peptides deviating in its sequence from the wt PrAMP may possess similar activity. Previous studies of synthetic peptides with individual amino acid substitutions or screening of the limited (300–400 variants) single-codon substituted gene libraries of the only known Type II PrAMPs Api and Dro showed that some deviations from the wt sequences are allowed (15,22), but whether multiple amino acid replacements would preserve the peptides’ ability to act upon the terminating ribosome remained unknown. By analyzing a multi-substituted Api gene library, whose complexity approached 350,000 encoded peptide sequences, and by applying orthogonal screening strategies, we not only significantly expanded the variety of peptides acting upon the terminating ribosome, but also identified some unexpected trends in their structure-activity relationship.

Although we designed the library to have an increased mutation frequency towards the C-terminal segment of the peptide (Figure 1C), the active peptides showed a strong tendency to maintain the wild-type identity of the last 8 amino acid residues (Figure 4B, Supplementary Figure S5), underscoring the importance of this segment for the ribosome-targeting activity. The previous studies of the singly-substituted Api mutants had highlighted the functional significance of a shorter Api segment, P_14_-R_17_, which is a part of the P_11_-L_18_ sequence identified as critically important in the current studies. The difference in the results is not surprising because screening of the multi-substituted Api library was carried out under conditions of a milder expression of the endogenous Api gene in comparison with the previous experiments (22). Hence, the screening protocol used in the current studies selected against the mutations that even moderately interfere with the PrAMP’s ability to inhibit translation and cell growth, thereby preferentially identifying the most active peptides. Furthermore, in the multi-substituted library, a mildly negative effect of some of the C-terminal mutations maybe was exacerbated by additional amino acid alterations elsewhere in the peptide.

While the C-terminus of Api is generally intolerant to mutations, some substitutions are still allowed, including replacement of P_14_ or H_15_ with tyrosine or of P_16_ with leucine (Figure 4B). Corroborating these observations, some of the tested peptides with amino acid substitutions within the C-terminal segment could readily arrest the ribosome at the stop codon in the cell-free assays (Figures 2C and 3C). Several of the C-terminal residues of Api make direct contacts with rRNA in the NPET (8). Some of the tolerated substitutions could establish similar interactions with the ribosome. Thus, for example, the stacking interaction of the H15 side chain upon the guanine base of the 23S rRNA residue 2505 (8) may be preserved when this histidine residue is replaced with tyrosine. The other substitutions could possibly generate alternative contacts with the NPET sufficient for maintaining the affinity of the peptide for its binding site.

Contrasting the invariance of the C-terminal segment, multiple alterations are allowed within the N-terminal half of the Api sequence. Nevertheless, the N-terminal portion is functionally important and likely modulates the activity of the endogenously expressed PrAMP. This conclusion follows from the prevalence of specific amino acid residues at defined positions within the N-terminal segment. Counterintuitively, however, some of the wt residues (e.g. P_5_, I_8_ or Q_10_) are selected against in the active peptides. It is possible that the identities of these residues in wt Api are important for the PrAMP uptake by the bacterial cell (an inconsequential metric for our selection which is based on endogenously expressed PrAMP) or they may support Api interactions with the ribosomes of the insect's bacterial pathogens but are not optimal for Api's action upon the *E. coli* ribosome. The first three amino acids (G_1_N_2_N_3_) of Api are also frequently found replaced in the endogenously expressed active peptides (Figure 4B). Either mutations of these residues enhance the on-target activity of the encoded peptides or alterations of the respective mRNA codons that are proximal to the translation start site increase the efficiency of expression of the Api gene.

Two orthogonal strategies were used in our experiments: the first was based on the negative selection involving depletion of the clones expressing active peptides, while the other one took advantage of the preferential survival of the clones expressing PrAMPs conducive to stop codon readthrough. While the general sequence trends of the active peptides that were identified by both selection strategies were similar (compare Figure 4B and Supplementary Figure S5), some notable differences emerged such as, for example, a stronger preference for retaining all the Api's proline residues in the readthrough-inducing peptides (Figure 4C). We can only speculate what drives these differences. The known effects of Api on translation include the arrest of the post-release ribosome at stop codons, queuing of the translating ribosomes behind the arrested one, and RF trapping leading to stop codon readthrough and accumulation of aberrant proteins with C-terminal extensions (8,13). Cell growth inhibition, which is the main discriminating criterion in the negative selection experiments, is likely a product of all these effects of the PrAMP and, possibly, of others yet unknown. In contrast, our positive selection strategy relied specifically on the ability to cause stop codon readthrough. The idiosyncratic sequence preferences of the readthrough-inducing peptides may reflect their propensity for a more prolonged RF trapping possibly because of a slower resolution of the ribosome-Api-RF complex (12).

Conceivably, carrying out orthogonal selection strategies not in parallel, as was done in this study, but sequentially would increase the stringency of selection. Enabling our positive selection for high-throughput screening and supplementing it subsequently by negative selection could more effectively eliminate the false-positive peptide sequences and, hence, make the results more robust. This approach could be implemented in future studies of Type II PrAMPs and other inhibitors with mechanisms of action akin to that of Api.

As a collateral result of our effort in identifying the spectrum of ribosome-targeting peptides, our selection strategies also yielded new PrAMPs with antibacterial activity. Several of the selected multi-substituted peptides were more active than Api in inhibiting *E. coli* growth. We noted that among the tested peptides identified by the alternative selection strategies, a larger fraction of PrAMPs with pronounced antibacterial activity was found among those that emerged from the positive selection that identified clones able to survive due to the PrAMP-dependent stop codon readthrough. It appears that the positive selection approaches could be beneficial for identifying peptides with antibiotic properties. Furthermore, analogous screening of Api mutant gene libraries expressed in clinically relevant hosts may identify PrAMP variants better suited for development of therapeutically active agents than wt Api.

While elucidating the antibacterial activity of PrAMPs has obvious medical significance, understanding the mechanistic aspects of their action may also provide important insights into fundamental aspects of translation. The constellation of the NPET-bound PrAMP, deacylated P-site tRNA and the A-site associated RF strikingly resembles the state of the translating ribosome immediately after the hydrolysis of the peptidyl-tRNA ester bond. The structural similarity of the Api-ribosome complex with the native post-release ribosome raises the possibility that the C-termini of some cellular proteins may also linger in the NPET after peptidyl-tRNA hydrolysis, possibly also transiently trapping RFs, slowing down their dissociation and the ribosome recycling. In such a scenario, the cell inhibitory action of Type II PrAMPs could be the extreme manifestation of a more general phenomenon of the lingering of the nascent chain in the NPET after peptidyl-tRNA hydrolysis. Our exploration of the multi-substituted Api libraries illuminates the possibility that many polypeptide sequences, exhibiting different degree of similarity with the antimicrobial PrAMP could be transiently retained in the ribosomal NPET after completion of their translation. The delayed protein release could be exploited by the cell for improved protein folding, targeting, or interaction with cellular ligands.

## Supplementary Material

gkae567_Supplemental_Files

## Data Availability

The NGS data are available at GEO with the accession number GSE269894.

## Notes

Present address: Chetana Baliga, Department of Biotechnology, Faculty of Life and Allied Health Sciences M.S. Ramaiah University of Applied Sciences, New BEL Road, MSR Nagar, Bangalore 560054, Karnataka, India.
