# Supplementary material for: Sequence diversity of apidaecin-like peptides arresting the terminating ribosome
Source: Nucleic Acids Res. 2024 Jul 2;52(15):8967–78. doi: 10.1093/nar/gkae567 (PMC11347161; doi:10.1093/nar/gkae567)
Supplement: gkae567_Supplemental_Files [file gkae567_supplemental_files.zip › Supplementary Info.revised.docx]

**Sequence diversity of apidaecin-like peptides arresting the terminating ribosome**

Weiping Huang, Chetana Baliga, Alexander S. Mankin, Nora Vázquez-Laslop

**Supplementary Information**

**Figures and Tables**

**Supplementary Table S1. The frequency of the codons used for replacement of the wt Api gene codons during massively parallel synthesis of the multi-substituted Api gene library**

| **Amino acid** | **Codon** | **Frequency** |  | **Amino acid** | **Codon** | **Frequency** |
| --- | --- | --- | --- | --- | --- | --- |
| Ala | GCA | 28% |  | Pro | CCA | 33% |
| Ala | GCC | 24% |  | Pro | CCC | 16% |
| Ala | GCG | 18% |  | Pro | CCG | 25% |
| Ala | GCT | 30% |  | Pro | CCT | 28% |
| Cys | TGC | 43% |  | Gln | CAA | 52% |
| Cys | TGT | 58% |  | Gln | CAG | 48% |
| Asp | GAC | 35% |  | Arg | AGA | 58% |
| Asp | GAT | 65% |  | Arg | AGG | 0% |
| Glu | GAA | 67% |  | Arg | CGA | 0% |
| Glu | GAG | 33% |  | Arg | CGC | 0% |
| Phe | TTC | 39% |  | Arg | CGG | 0% |
| Phe | TTT | 62% |  | Arg | CGT | 42% |
| Gly | GGA | 21% |  | Ser | AGC | 16% |
| Gly | GGC | 24% |  | Ser | AGT | 17% |
| Gly | GGG | 15% |  | Ser | TCA | 20% |
| Gly | GGT | 41% |  | Ser | TCC | 15% |
| His | CAC | 37% |  | Ser | TCG | 11% |
| His | CAT | 64% |  | Ser | TCT | 22% |
| Ile | ATA | 24% |  | Thr | ACA | 28% |
| Ile | ATC | 29% |  | Thr | ACC | 27% |
| Ile | ATT | 47% |  | Thr | ACG | 18% |
| Lys | AAA | 65% |  | Thr | ACT | 29% |
| Lys | AAG | 36% |  | Val | GTA | 20% |
| Leu | CTA | 0% |  | Val | GTC | 20% |
| Leu | CTC | 0% |  | Val | GTG | 24% |
| Leu | CTG | 30% |  | Val | GTT | 36% |
| Leu | CTT | 17% |  | Trp | TGG | 100% |
| Leu | TTA | 28% |  | Tyr | TAC | 40% |
| Leu | TTG | 25% |  | Tyr | TAT | 61% |
| Met | ATG | 100% |  | * | TAA | 53% |
| Asn | AAC | 41% |  | * | TAG | 16% |
| Asn | AAT | 59% |  | * | TGA | 32% |

**Supplementary Table S2. DNA primers used for preparation of the next-generation sequencing libraries**

| **Screening method** | **Group** | **Primer Name** | **Sequences** |
| --- | --- | --- | --- |
| **Depletion**  **Selection** | Arabinose replicate 1 | Api_Lib_F_i56 | AATGATACGGCGACCACCGAGATCTACACCCTTGTAGACACTCTTTCCCTACACGACGCTCTTCCGATCTCTAGAACTAGTAGGAGGAT |
|  |  | Api_Lib_R_i76 | CAAGCAGAAGACGGCATACGAGATTTCCAAGGGGTGACTGGAGTTCAGACGTGTGCTCTTCCGATCTAAGCGTGACATAACTAATT |
|  | Arabinose replicate 2 | Api_Lib_F_i54 | AATGATACGGCGACCACCGAGATCTACACTAACCGGTACACTCTTTCCCTACACGACGCTCTTCCGATCTCTAGAACTAGTAGGAGGAT |
|  |  | Api_Lib_R_i74 | CAAGCAGAAGACGGCATACGAGATGAGACGATGTGACTGGAGTTCAGACGTGTGCTCTTCCGATCTAAGCGTGACATAACTAATT |
|  | Arabinose replicate 3 | Api_Lib_F_i58 | AATGATACGGCGACCACCGAGATCTACACGTTCTCGTACACTCTTTCCCTACACGACGCTCTTCCGATCTCTAGAACTAGTAGGAGGAT |
|  |  | Api_Lib_R_i78 | CAAGCAGAAGACGGCATACGAGATACGGAACAGGTGACTGGAGTTCAGACGTGTGCTCTTCCGATCTAAGCGTGACATAACTAATT |
|  | Glucose replicate 1 | Api_Lib_F_i57 | AATGATACGGCGACCACCGAGATCTACACTCAGGCTTACACTCTTTCCCTACACGACGCTCTTCCGATCTCTAGAACTAGTAGGAGGAT |
|  |  | Api_Lib_R_i77 | CAAGCAGAAGACGGCATACGAGATCGCATGATGGTGACTGGAGTTCAGACGTGTGCTCTTCCGATCTAAGCGTGACATAACTAATT |
|  | Glucose replicate 2 | Api_Lib_F_i55 | AATGATACGGCGACCACCGAGATCTACACGAACATCGACACTCTTTCCCTACACGACGCTCTTCCGATCTCTAGAACTAGTAGGAGGAT |
|  |  | Api_Lib_R_i75 | CAAGCAGAAGACGGCATACGAGATCTTGTCGAGTGACTGGAGTTCAGACGTGTGCTCTTCCGATCTAAGCGTGACATAACTAATT |
|  | Glucose replicate 3 | Api_Lib_F_i59 | AATGATACGGCGACCACCGAGATCTACACAGAACGAGACACTCTTTCCCTACACGACGCTCTTCCGATCTCTAGAACTAGTAGGAGGAT |
|  |  | Api_Lib_R_i79 | CAAGCAGAAGACGGCATACGAGATCGGCTAATGGTGACTGGAGTTCAGACGTGTGCTCTTCCGATCTAAGCGTGACATAACTAATT |
| **Positive selection** | Arabinose sample | Api_Lib_F_i55 | AATGATACGGCGACCACCGAGATCTACACGAACATCGACACTCTTTCCCTACACGACGCTCTTCCGATCTCTAGAACTAGTAGGAGGAT |
|  |  | Api_Lib_R_i75 | CAAGCAGAAGACGGCATACGAGATCTTGTCGAGTGACTGGAGTTCAGACGTGTGCTCTTCCGATCTAAGCGTGACATAACTAATT |

**
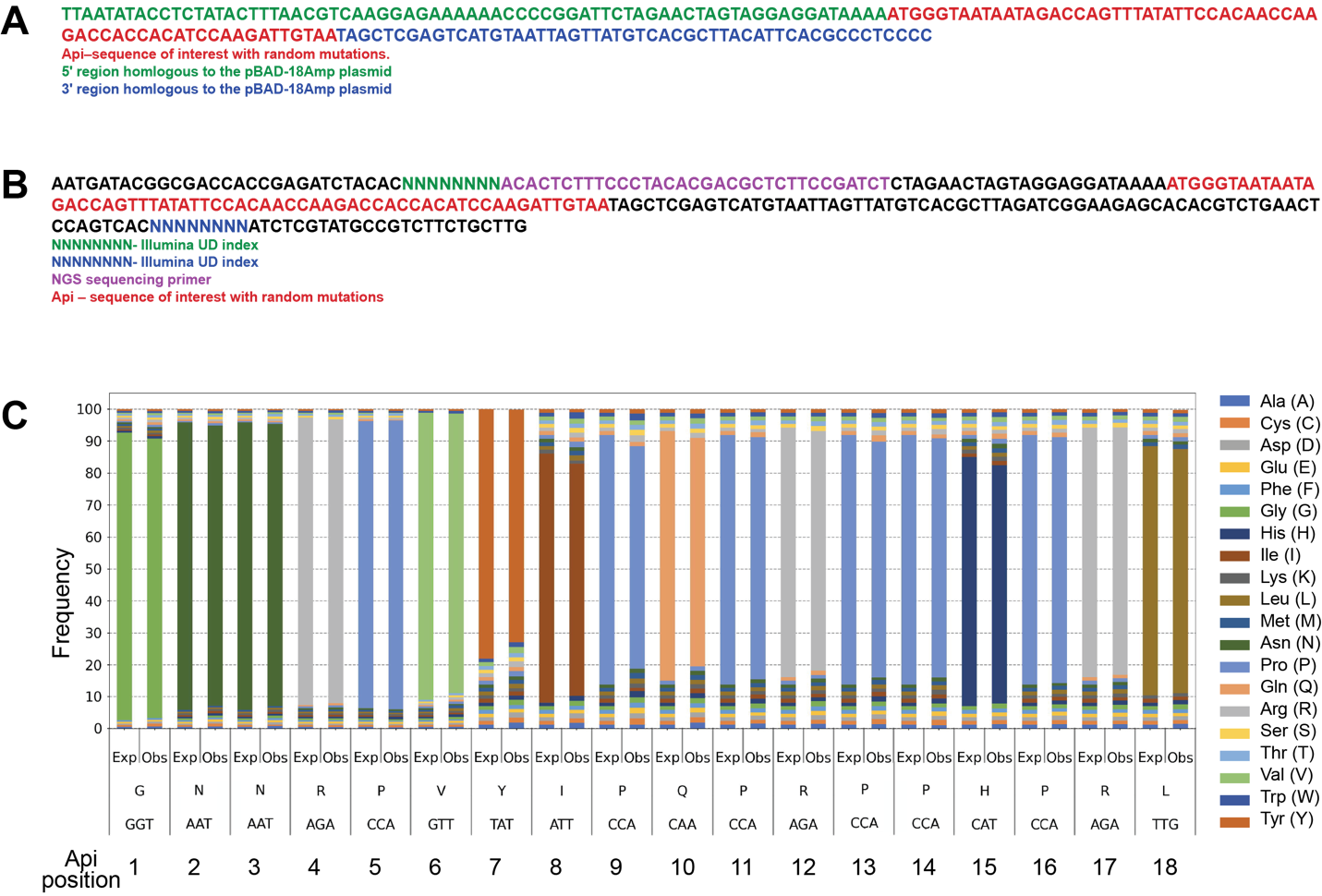
**

**Supplementary Figure S1. *The library encoding multi-substituted Api peptide mutants***. **A**, The structure of the synthetic DNA fragment library used for generation of the plasmid library by Gibson assembly with the pBAD vector. Shown in red is the wt sequence of Api which was mutated during DNA synthesis. **B**, The structure of the DNA fragments amplified from the plasmid library for NGS. **C**, Expected (‘Exp’) and observed (‘Obs’) distribution of codons specifying individual amino acids in the synthetic DNA fragment library prepared by massively parallel synthesis and analyzed by NGS prior to cloning in the expression vector.

|  | **1** | **2** | **3** | **4** | **5** | **6** | **7** | **8** | **9** | **10** | **11** | **12** | **13** | **14** | **15** | **16** | **17** | **18** |
| --- | --- | --- | --- | --- | --- | --- | --- | --- | --- | --- | --- | --- | --- | --- | --- | --- | --- | --- |
| **M** | 0.35% | 0.38% | 0.38% | 0.38% | 0.40% | 0.68% | 0.94% | 1.13% | 1.66% | 1.25% | 1.33% | 1.26% | 1.29% | 1.33% | 1.19% | 0.99% | 1.18% | 1.06% |
| **A** | 0.39% | 0.53% | 0.48% | 0.27% | 0.26% | 0.46% | 1.36% | 1.37% | 0.96% | 1.65% | 1.31% | 1.27% | 1.13% | 0.87% | 1.14% | 1.05% | 1.09% | 1.15% |
| **C** | 0.40% | 0.54% | 0.36% | 0.36% | 0.52% | 0.48% | 1.28% | 1.14% | 1.96% | 1.46% | 1.11% | 1.13% | 1.43% | 1.73% | 1.04% | 1.15% | 0.87% | 0.96% |
| **D** | 0.38% | 0.39% | 0.50% | 0.39% | 0.32% | 0.38% | 1.30% | 1.12% | 1.36% | 1.38% | 1.13% | 1.23% | 1.22% | 1.19% | 0.95% | 1.10% | 0.97% | 1.00% |
| **E** | 0.38% | 0.56% | 0.43% | 0.52% | 0.49% | 0.45% | 0.85% | 0.85% | 1.76% | 1.20% | 0.85% | 1.11% | 1.14% | 1.30% | 1.04% | 1.03% | 1.00% | 0.73% |
| **F** | 0.26% | 0.30% | 0.40% | 0.23% | 0.21% | 0.30% | 0.85% | 1.04% | 1.68% | 1.38% | 1.17% | 1.23% | 1.24% | 1.15% | 1.07% | 0.94% | 0.95% | 0.87% |
| **G** | 92.84% | 0.45% | 0.55% | 0.36% | 0.33% | 0.52% | 1.28% | 1.20% | 1.58% | 1.40% | 1.34% | 1.45% | 1.26% | 1.11% | 1.27% | 1.16% | 1.11% | 1.25% |
| **H** | 0.54% | 0.44% | 0.39% | 0.39% | 0.35% | 0.56% | 0.99% | 1.17% | 1.36% | 1.16% | 1.18% | 1.13% | 1.16% | 1.25% | 79.30% | 0.89% | 1.03% | 1.00% |
| **I** | 0.27% | 0.44% | 0.38% | 0.40% | 0.21% | 0.44% | 1.16% | 78.82% | 0.94% | 1.18% | 1.25% | 0.96% | 1.32% | 1.22% | 1.13% | 0.89% | 0.81% | 0.85% |
| **K** | 0.28% | 0.45% | 0.48% | 0.33% | 0.36% | 0.40% | 0.73% | 0.74% | 1.36% | 1.23% | 0.94% | 0.91% | 0.81% | 1.56% | 1.08% | 0.76% | 1.01% | 0.87% |
| **L** | 0.41% | 0.33% | 0.39% | 0.39% | 0.38% | 0.43% | 1.19% | 1.31% | 1.37% | 1.20% | 1.08% | 1.00% | 1.29% | 1.31% | 1.06% | 1.14% | 0.94% | 81.83% |
| **N** | 0.28% | 91.84% | 92.24% | 0.46% | 0.35% | 0.31% | 0.81% | 0.83% | 1.26% | 1.12% | 1.04% | 1.03% | 0.71% | 1.13% | 1.12% | 0.77% | 0.94% | 0.89% |
| **P** | 0.48% | 0.48% | 0.28% | 0.42% | 93.07% | 0.60% | 1.24% | 1.36% | 72.02% | 1.61% | 78.92% | 1.00% | 77.50% | 76.49% | 1.09% | 81.47% | 0.92% | 1.00% |
| **Q** | 0.45% | 0.36% | 0.30% | 0.45% | 0.18% | 0.51% | 0.96% | 1.06% | 1.24% | 74.98% | 1.14% | 1.20% | 1.24% | 1.13% | 1.30% | 0.91% | 0.91% | 0.95% |
| **R** | 0.42% | 0.46% | 0.43% | 92.60% | 0.54% | 0.53% | 1.40% | 1.16% | 1.98% | 1.31% | 0.88% | 78.15% | 1.32% | 1.53% | 0.97% | 0.94% | 81.73% | 0.93% |
| **S** | 0.42% | 0.43% | 0.51% | 0.34% | 0.52% | 0.43% | 0.98% | 1.09% | 1.62% | 1.11% | 1.04% | 1.19% | 1.10% | 1.36% | 0.82% | 1.03% | 1.04% | 0.89% |
| **T** | 0.46% | 0.38% | 0.43% | 0.43% | 0.51% | 0.58% | 1.06% | 1.10% | 1.14% | 1.42% | 1.14% | 1.24% | 1.17% | 0.87% | 1.13% | 0.98% | 0.85% | 0.94% |
| **V** | 0.31% | 0.36% | 0.41% | 0.53% | 0.23% | 90.87% | 1.44% | 1.14% | 1.36% | 1.43% | 1.04% | 1.42% | 1.20% | 0.86% | 1.28% | 1.00% | 0.99% | 1.00% |
| **W** | 0.32% | 0.48% | 0.37% | 0.47% | 0.36% | 0.65% | 1.17% | 1.61% | 2.01% | 1.27% | 1.19% | 1.06% | 1.39% | 1.33% | 1.29% | 1.03% | 0.93% | 0.90% |
| **Y** | 0.34% | 0.38% | 0.29% | 0.26% | 0.40% | 0.44% | 79.00% | 0.77% | 1.37% | 1.26% | 0.92% | 1.03% | 1.10% | 1.26% | 0.75% | 0.78% | 0.74% | 0.93% |

**Supplementary Figure S2. *Frequency of amino acid substitutions at individual positions of the peptides encoded in the mutant api gene library.*** Wild type amino acids are indicated in green.

**
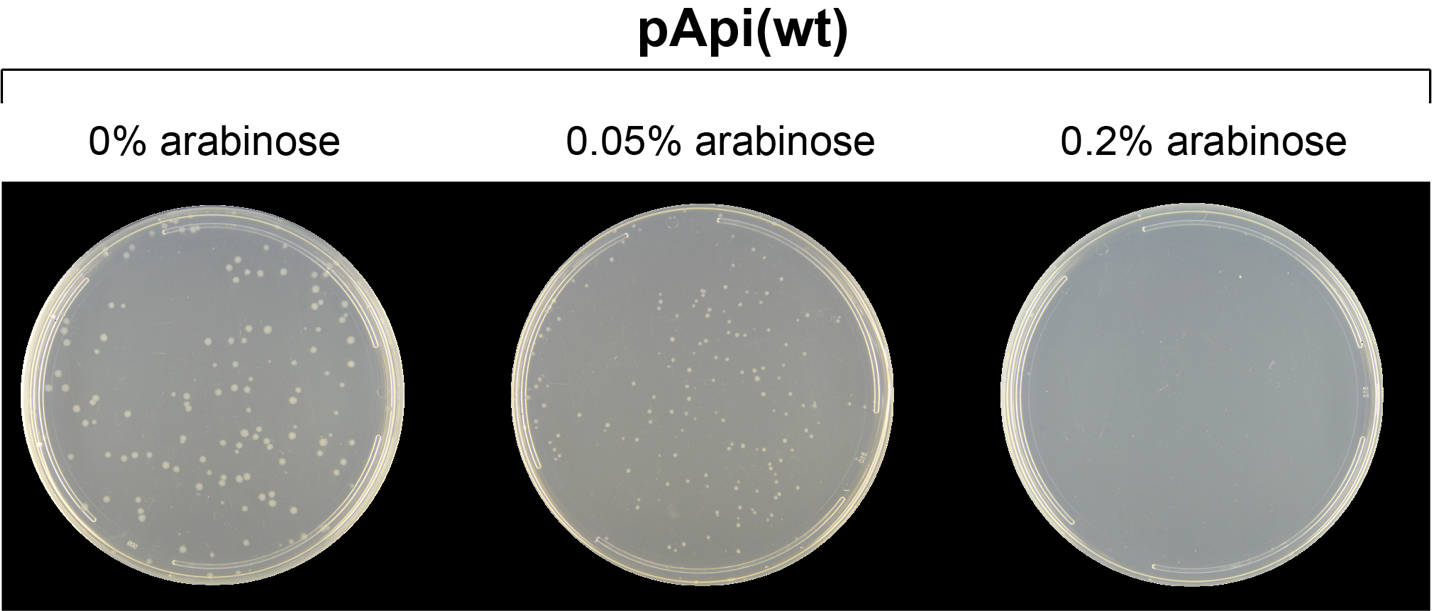
**

**Supplementary Figure S3. *Optimizing the level of expression of the peptides in the cells transformed with the multi-substituted Api gene library*.** *E. coli* BL21 cells transformed with the pApi plasmid encoding wt Api plated onto LB/Amp plates with varying concentration of arabinose, the inducer of the *api* gene expression.

**
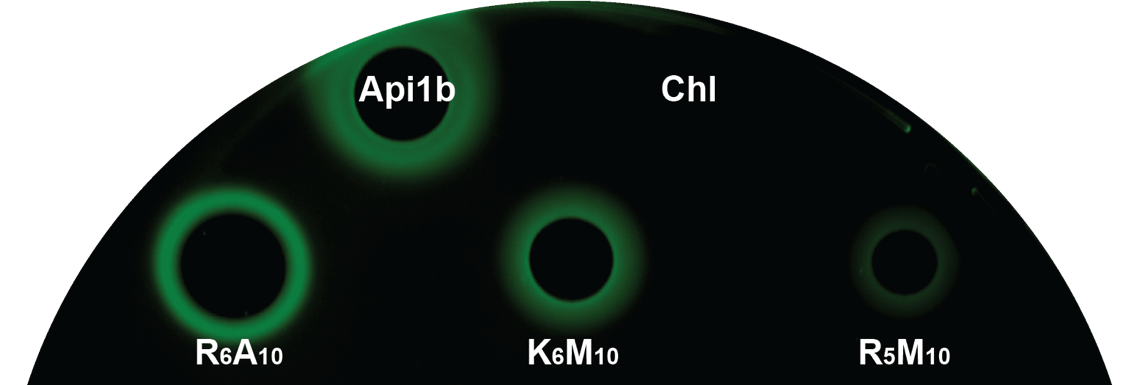
**

**Supplementary Figure S4. *Peptides with antibacterial activity that originated from the depletion selection can induce stop codon readthrough***. For experimental details, see Figure 3D legend. Chloramphenicol (Chl), an antibiotic that does not induce stop codon readthrough, was used as a negative control.

**
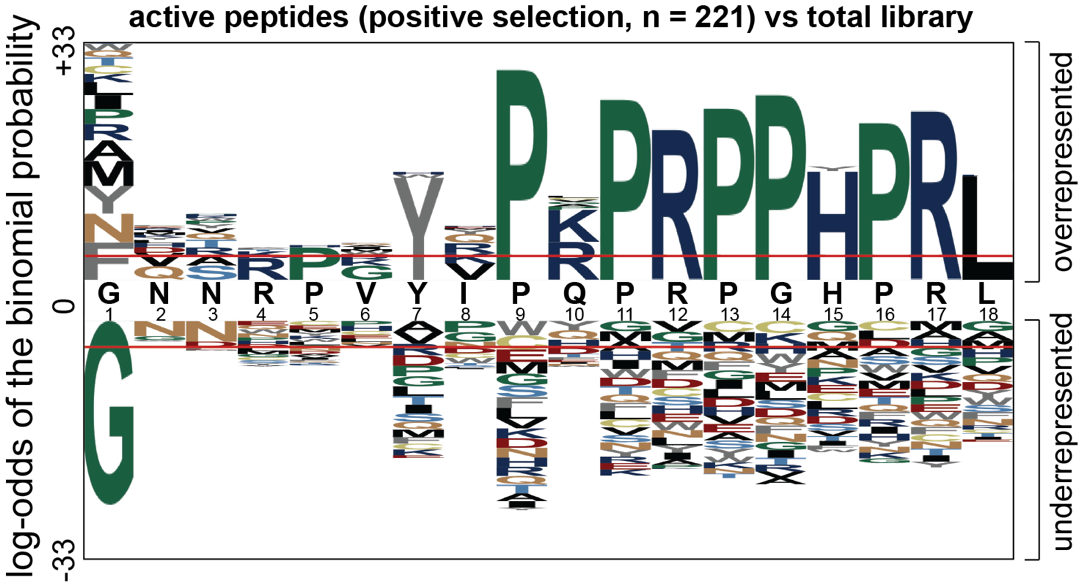
**

**Supplementary Figure S5. *pLogo analysis of amino acid enrichment at individual positions of the active peptides identified by the positive selection in comparison with the unselected peptides.*** Preferred amino acids appear in the upper panel (overrepresented); the amino acids that are counter selected in the active peptides appear in the bottom panel (underrepresented).


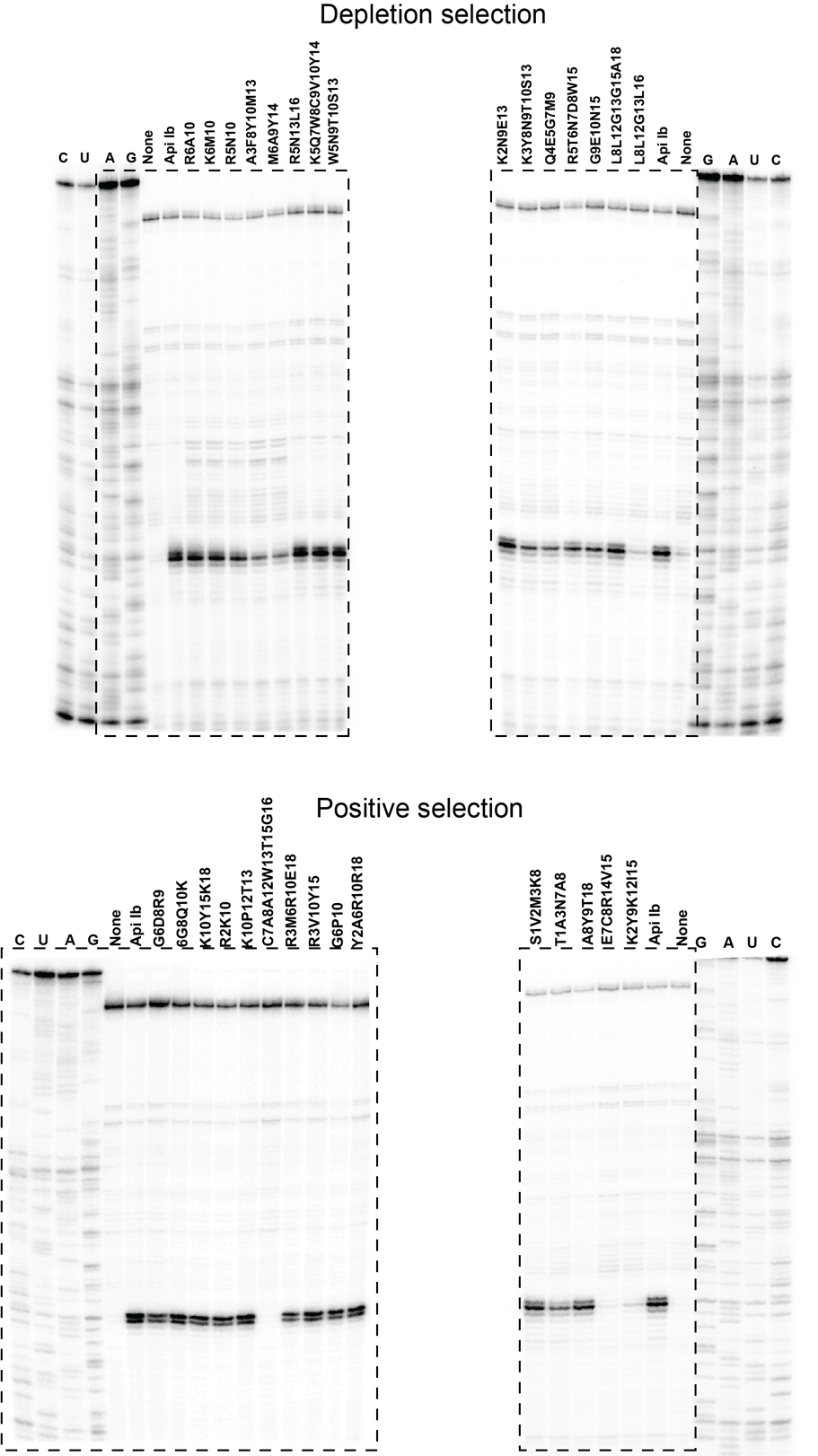


**Supplementary Figure S6. *Uncropped toeprinting gels shown in Figures 2D and 3D.***
